# Tertiary lymphoid structures in ovarian cancer

**DOI:** 10.3389/fimmu.2024.1465516

**Published:** 2024-11-06

**Authors:** Guojuan Sun, Yi Liu

**Affiliations:** ^1^ The Ward Section of Home Overseas Doctors, Hospital of Chengdu University of Traditional Chinese Medicine, Chengdu, China; ^2^ Department of Gynaecology and Obstetrics, Sichuan Provincial People’s Hospital, School of Medicine, University of Electronic Science and Technology of China, Chengdu, China

**Keywords:** tertiary lymphoid structures, ovarian cancer, tumor microenvironment, immune environment, immunotherapies

## Abstract

Ovarian cancer (OC) is a significant cause of cancer-related mortality in women worldwide. Despite advances in treatment modalities, including surgery and chemotherapy, the overall prognosis for OC patients remains poor, particularly for patients with advanced or recurrent disease. Immunotherapy, particularly immune checkpoint blockade (ICB), has revolutionized cancer treatment in various malignancies but has shown limited efficacy in treating OC, which is primarily attributed to the immunologically. Tertiary lymphoid structures (TLSs), which are ectopic aggregates of immune cells, have emerged as potential mediators of antitumor immunity. This review explores the composition, formation, and induction of tumor associated TLS (TA-TLS) in OC, along with their role and therapeutic implications in disease development and treatment. By elucidating the roles TA-TLSs and their cellular compositions played in OC microenvironment, novel therapeutic targets may be identified to overcome immune suppression and enhance immunotherapy efficacy in ovarian cancer.

## 1 Introduction

Ovarian cancer remains one of the most lethal gynecologic malignancies and is characterized by late-stage diagnosis and high recurrence rates (1, 2). Despite advances in surgical techniques and chemotherapeutic regimens, the prognosis for ovarian cancer patients remains poor, necessitating the exploration of novel therapeutic approaches (3). One promising area of research is the immune microenvironment of ovarian tumors, which plays a crucial role in tumor progression and patient outcomes. The immune microenvironment in ovarian cancer is a complex network of immune cells, cytokines, and chemokines that interact with tumor cells to influence disease progression. Understanding the tumor microenvironment (TME) is essential for developing effective immunotherapies.

Traditional immunotherapy research often focuses on monocellular populations in TME. In recent years, immune therapies have focused heavily on CD8+ cytotoxic T lymphocytes (CTLs). Within this area, chimeric antigen receptor T-cell (CAR-T) therapy, T-cell receptor T-cell (TCRT) therapy and ICB therapy have achieved tremendous success and revolutionized cancer treatment (4). However, under the shadow of immune therapies, the role of the humoral immune response has long been overlooked. Many studies have shown that compared to the elevated infiltration of CD8+ CTLs, the co-infiltration of B and T cells, which is indicative of the presence of TLSs, is associated with increased survival in patients with ovarian cancer (5, 6).

Tertiary lymphoid structures (TLSs) are ectopic lymphoid structures that resemble secondary lymphoid organs but arise in nonlymphoid tissues during chronic inflammatory diseases, including cancer, infections, autoimmunity and aging (7, 8). TLSs were initially documented in inflammatory diseases. For example, TLSs triggered by infections elicit beneficial antipathogen immune responses in the host (9). Conversely, in autoimmune diseases, such as rheumatoid arthritis, Sjögren syndrome TLSs facilitate the activation of autoreactive lymphocytes, leading to the production of autoantibodies, and their presence is correlated with an unfavorable prognosis (10, 11). Recent studies have highlighted the significance of tumor-associated tertiary lymphoid structures (TA-TLSs) in the TME.

Numerous studies have demonstrated that TA-TLSs can provide a specialized immune niche for T/B-cell infiltration and proliferation, enhancing local antitumor immunity by promoting interactions between cellular and humoral immunity (12, 13). The presence of TA-TLSs has been associated with favorable prognosis in various cancers, including ovarian cancer (14–17). Given the complexity of TLS biology, deeper exploration of TLS formation, function, and detection within the TME is necessary. The importance of TA-TLSs and the crosstalk of lymphocytes within them has gained increasing attention. Therefore, investigating the function and characterization of TLSs in the TME is a crucial research direction for ovarian cancer immunotherapy.

Studies have shown that TA-TLSs and their constituents play significant roles in ovarian cancer prognosis and immune therapy. Commencing the cellular composition and function of TA-TLSs, we reviewed their roles in OC and summarized their identification and induction methods, aiming to provide reliable evidence for the study and application of TA-TLSs in ovarian cancer.

## 2 TLS formation and function in ovarian cancer

### 2.1 Mechanisms underlying TLS formation

TLSs are defined as ectopic lymphatic aggregates with similarities to secondary lymphoid organs (SLOs). TLSs are most commonly found in regions with inflamed environments, such as autoimmune diseases (18), transplanted organs (19), chronic inflammation (20), and tumor sites (7, 21). Although the specific composition of TLSs may vary among cancer types (22), T cells, B cells, dendritic cells (DCs), macrophages and stromal cells are usually observed (7). However, the mechanisms governing the formation, maintenance, and function of TLSs are not fully understood. It seems that a specific set of cells and chemokines orchestrates TLS formation. Due to the structural and functional similarities between TLS and SLO, it was previously assumed that they followed a similar formation mechanism. However, the detailed mechanisms and variations depending on the TME context remain unresolved.

SLO originates in embryonic lymphoid tissue through the interaction of a lymphoid tissue inducer cell (LTi) with a lymphoid tissue organizer cell (LTo). Usually, LTi cells express RORγt and Id2 and drive the initial steps of SLO formation by activating LTo cells (23). LTo cells in lymph nodes are mesenchymal cells that later differentiate into follicular dendritic cells (FDCs) and fibroblastic reticular cells (FRCs) in a tumor necrosis factor (TNF) family member, especially in a lymphotoxin-dependent manner (21). TLSs were observed in adult nonlymphoid tissues, which lack embryonic-derived LTi cells. Thus, with respect to the upstream initiation of TLSs, there is a major question: who flipped the switch? Current studies have suggested that LTi and LTo cells are alternative for TLSs. However, the exact origin of these cells in humans is not clear.

Several surrogate LTi and LTo cells have been observed in mouse models. Unlike the bona fide LTi cells in SLOs the surrogate LTi cells in TLS such as T cells, B cells, NK cells, macrophages may be attracted to the inflammatory site by CXCL13 and IL-7 from inflammatory microenvironment and activate potential LTo cells (mostly stromal or immune cells) in a TNF family receptor (LTR/TNFR)-dependent manner (7). In addition, TNF family-independent LTo activation was observed in murine lung TLSs. For instance, TLS formation in microbially stimulated murine lungs can be initiated by interleukin-17 (IL-17) derived from T cells by promoting LTα-independent CXC-chemokine ligands 13 (CXCL13) expression (24, 25). Unlike the bona fide LTi cells, innate lymphoid cell 3, in SLOs, the surrogate LTi cells in TLSs were found to be immune cells such as T cells and B cells (7).

**Figure 1:**
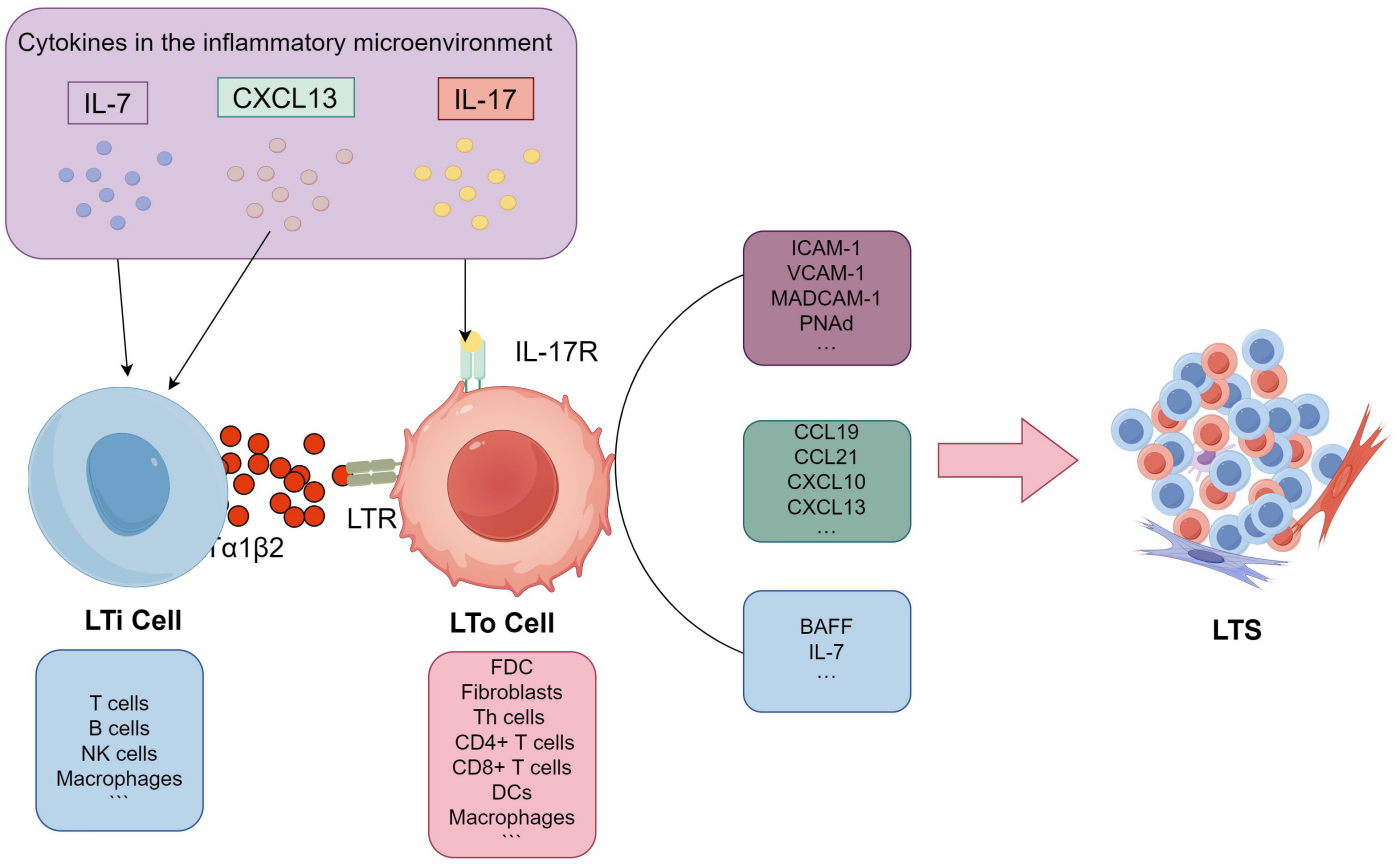
Potential pathways for TLS formation. Firstly, inflammatory chemokines like CXCL13 and IL-7 attract surrogate LTi cells to the site of inflammation within the inflammatory microenvironment. The surrogate LTi cells then activate potential LTo cells, which are primarily composed of stromal or immune cells, through either the LTα/TNF receptor-dependent (LT/LTR) or independent (IL-17) signaling pathways. Activated LTo cells subsequently produce a variety of lymphoid chemokines, including CCL19, CCL21, CXCL10, and CXCL13, as well as adhesion molecules such as VCAM-1, ICAM-1, MAdCAM-1, and PNAd. They also produce lymphocyte survival factors like BAFF and IL-7 to recruit lymphocytes and facilitate subsequent TLS formation. LT/LTR, lymphotoxin/lymphotoxin receptor; LTi, lymphoid tissue inducer cell (LTi); LTo, lymphoid tissue organizer cell; CCL19, CC-chemokine ligands 19; CCL21,CC-chemokine ligands21; CXCL10, CXC-chemokine ligands 10; CXCL13, CXC-chemokine ligands 13; VCAM-1,vascular cell-adhesion molecule 1; ICAM-1, intercellular adhesion molecule 1; MAdCAM-1,mucosal addressing cell-adhesion molecule 1; PNAd, peripheral node addressin; BAFF, B-cell activating factor; IL-7, interleukin 7; IL-17, interleukin 17.

In both SLOs and TLSs, LTo cells play a critical role in organizing the immune response. By producing a variety of chemokines, adhesion molecules, and survival factors, LTo cells help recruit and guide immune cells to the site of immune activity and facilitate vascularization, which supports the immune response (26, 27). The chemokines produced by LTo cells, including CC-chemokine ligands (CCL19 and CCL21), CXC-chemokine ligands (CXCL10 and CXCL13), are crucial for attracting different immune cells. These chemokines create gradients that guide these cells to specific locations within the lymphoid structures. Additionally, adhesion molecules such as vascular cell-adhesion molecule 1 (VCAM-1), intercellular adhesion molecule 1 (ICAM-1), mucosal addressing cell-adhesion molecule 1 (MAdCAM-1), and peripheral node addressin (PNAd) helps tether circulating immune cells and allows them to extravasate into the tissue. Finally, survival factors such as BAFF (B-cell activating factor) and IL-7 play roles in promoting the survival and maturation of B and T lymphocytes, which are critical for maintaining functional immune responses. In SLOs, the origins of LTo subsets are usually fixed. FDCs and fibroblasts are the most effective LTo cells (26). However, the number and type of LTo cells in TA-TLSs may vary by tumor types. Currently, immune cells and stromal cells such as Th cells (28) and fibroblasts (29) in breast cancer, fibroblasts in melanoma (30), CD8+ T cells (31), DCs (32) in lung cancer, and macrophages (33), CD4+ T cells, and DCs (17) in ovarian cancer have all been reported as potential LTo cells that secrete homeostatic chemokines. The classic LT/LTR dependent TLS formation pathway has been meticulously illustrated in **Figure 1**, showcasing the intricate processes and interactions involved in this well-established mechanism, which plays a crucial role in the understanding of cellular responses and adaptations.

Our understanding of TLS formation primarily stems from studies of autoimmune diseases and chronic inflammation. Although these studies offer valuable insights, they also highlight important knowledge gaps. Overall, the formation mechanism of TA-TLS still remains ambiguous. For instance, it is uncertain whether LTi and LTo cells are crucial for TA-TLS formation. Additionally, the specific cells responsible for these critical functions within the complex tumor microenvironment remain unidentified. This uncertainty presents researchers with numerous unanswered questions and avenues for further investigation.

### 2.2 The role TA-TLSs played in ovarian cancer

Traditionally, an efficient adaptive immune response against cancer that occurs in SLOs has been extensively documented and broadly accepted (34). However, studies on the tumor microenvironment further revealed that tumor-associated TA-TLSs are important immune response sites *in situ* and have been proven to exacerbate the local immune response in tumors and help promote an efficacious immune contexture (35). In patients with ovarian cancer, TA-TLS is associated with good prognosis, regardless of the presence or absence of vascular thrombosis and lymph node metastasis (3).

With T cells as the target, ICB therapy such as PD-1/PD-L1 antibody has been the most successful immunotherapy for decades. TA-TLSs have been proven to improve favorable prognosis and improve ICB outcomes in several solid tumors (36, 37). This improvement may result from promoted tumor-targeting effector and memory T-cell responses, along with facilitated coordinated antitumor responses of T cells and B cells aggregating in TLS (38, 39). Unlike the so-called “hot tumors,” such as non-small cell lung cancer (NSCLC) or melanoma, most ovarian cancers do not respond to ICB therapy most likely due to indolent anticancer immunity and active immunosuppression (40). In High-grade serous ovarian cancer (HGSOC), the most common and deadly type of ovarian cancer, several studies have shown a correlation between the presence of TA-TLSs and improved prognosis as well as a favorable response to ICB therapy (5, 6). Nevertheless, the molecular mechanism by which TA-TLS improves ICB outcomes in HGSOC is still not well understood.

As recent studies have shown that a high tumor mutation burden (TMB) predicts better ICB responsiveness in lung tumor patients (41). Kasikova et al. observed a greater abundance of TA-TLS in HGSOC with a higher TMB than in those with a lower TMB (42). Their further study indicated that the insensitivity of HGSOC to ICB therapy was linked to the limited number of mature TA-TLSs (mTLS) and ICB-sensitive TCF1+PD1+ CD8+ T cells. Thus, the low-to-intermediate TMB in ovarian cancer (43) and the resulting decrease frequent and development of TA-TLSs (42) may contribute to their poor response to ICB. These findings suggest that the targeted induction of TA-TLS holds significant promise in enhancing the efficacy of ICB therapy specifically in HGSOC, potentially leading to improved patient outcomes and a more robust therapeutic response.

TA-TLSs predominantly govern antitumor immunity through their cellular and molecular constituents. Although the specific composition of TA-TLSs may vary among cancer types, CD20+ B cells and CD3+ T cells make up the majority of TLSs (22). B cells and T cells are recruited to the tumor site by specific chemokines, where they organize into distinct zones within the TLS, similar to the architecture of secondary lymphoid organs. The core part of TLSs is the B-cell follicle, within which germinal centers (GCs) provide B cells with the ability to undergo somatic hypermutation, affinity maturation, and class switching, resulting in the generation of high-affinity antibodies. Disruption of GC formation may impair the prognostic value of TA-TLS (44). An increasing number of clinical studies have demonstrated that a high density of TA-TLSs and B cells, as well as the antitumor antigens they secrete, is associated with favorable disease outcomes not only in primary and metastatic OC (38, 45–47).

Around B-cell follicles, there is a T-cell zone that is involved in the activation and regulation of T-cell responses within TLSs. Several studies have confirmed that OC patients with greater T-cell infiltration in tumors experience significantly better overall survival (OS) (48, 49). However, recent research has suggested that the prognostic benefit of T cells in OC patients only exists in the presence of other cells in TLSs (38). Moreover, compared to the infiltration of T cells, the presence of TA-TLSs was associated with increased survival rates in patients with ovarian cancer (5, 6). This may be due to the ability of TA-TLS to infiltrate and expand T and B-cell lineages and enhance antitumoral immune responses by improving the interplay between cellular and humoral immunity.

Furthermore, as complex aggregates of leukocytes and specialized stromal cells, TLSs are not encapsulated and lack an independent vascular network requiring a stromal cell network to anchor them to chronically inflamed tissue sites (50). High endothelial venules (HEVs) formed by peripheral node addressin (PNAd)-positive endothelial cells (51) control the rate and type of lymphocytes recruited to TLSs (52). In the next part of this review, we will further elaborate on the roles of the cellular components of TA-TLSs in ovarian cancer.

## 3 Detection and evaluation of TA-TLSs in ovarian cancer

### 3.1 Traditional and innovative quantification method

**Figure 2:**
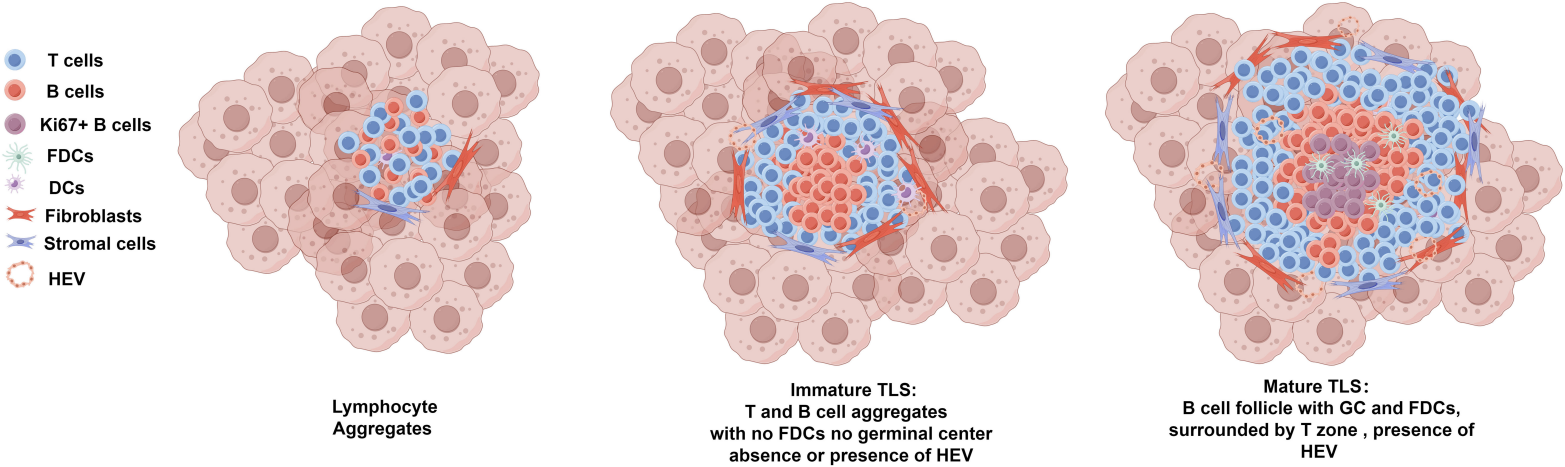
Morphological and compositional heterogeneous of TA-TLS in OC. Ranging from loose T-cell–B-cell clusters (lymphocyte aggregates) to highly organized mature TLS, TA-TLSs in OC are heterogeneous morphologically and compositionally. Lymphocyte aggregates typically refer to loose and disorganized clusters of T-cell–B-cells. Immature TLS usually refers to larger clusters of T/B lymphocyte aggregates that are anchored in inflammatory sites by fibroblasts and other stromal cells. Immature TLSs usually have B cell zone which generally located at the center of the TLS and surrounding T cell zone, but lacking CD21+ FDCs and GCs. Immature TLS can develop into mature TLS, which are generally larger morphologically. Mature TLS have prominent T/B cell zones, with the B cell zone containing GCs which characterized by Ki67+ proliferating B cells and an FDC network. As the specialized vasculature facilitates the entry of lymphocytes from the bloodstream into TLSs, HEVs can be observed in both immature and matured TLSs. TLSs, tertiary lymphoid structures; GCs, germinal centers; FDCs, follicular DCs; HEVs, high endothelial venules.

Ranging from loose T-cell–B-cell clusters to highly organized structures with distinct T-cell zones and B-cell follicles containing GCs, the heterogeneity of TLSs in terms of their cellular composition and spatial organization adds another layer of complexity, making it challenging to standardize their evaluation. As illustrated in **Figure 2**, the morphological and compositional heterogeneous of TA-TLS in OC underscores the intricate nature of these structures within the tumor microenvironment, revealing their diverse characteristics and functions. Furthermore, the role of TA-TLSs is not static, it fluctuates dynamically both within individual and across different cancers, reflecting the intricate interplay of immune responses (6, 53, 54). These discrepancies in the understanding and assessment of TLSs may arise from variations in how researchers define and detect these structures, leading to inconsistencies in the literature and complicating comparative studies across different research efforts.

In most previous studies, the quantification of TLSs was based on the morphological scale. The most straightforward morphology approach for identifying TLSs is pathological section counting with HE staining (22). In most cases, TLSs were defined as lymphoid aggregates according to HE staining without size limitations. However, in some TA-TLS-associated studies, lymphoid aggregates that were too small were defined as aggregates (AGGs). Currently, distinguishing between TLSs and small AGGs remains challenging (55). Thus, in some TA-TLS-associated studies, lymphocyte aggregates that were too small or had fewer than 200 cells were excluded to rule out AGGs (56, 57). Moreover, the identification of TLSs in HE-stained sections relies heavily on the experience of pathologists. The potential subjective differences may have led to the large differences in the study results. For instance, Kasikova et al. defined TLSs as lymphocyte aggregates via HE staining and observed TLSs in 155/209 (74%) HGSOC tumor specimens (42). However, with the same identification method, HE staining, Zhang et al. (39%, 29/74) (5) and Hou et al. (36.67%, 31/60) (6) revealed a significantly lower proportion of TA-TLSs in OC samples. Differences in cohort size may be one of the significant factors that results in discrepancies, but the influence of pathologists’ individual experience cannot be ruled out. Therefore, while HE-based TLS identification may appear straightforward, it is still too labor intensive and can be used for practical purposes (58). For this purpose, several automated computational workflows have been developed to quantify TLS density with HE-stained slides which could steer clinical trials in precision medicine by enhancing patient stratification (59–61).

To improve evidence, immunohistochemistry (IHC) and immunofluorescence (IF) are usually used for TLS screening and maturity detection, which makes biomarkers of TLS detection and maturation crucial. Although the specific composition of TA-TLSs may vary among cancer types, biomarkers such as CD20, CD3, CD8, PNAd, and LAMP are common across different tumors (22). Different TLS criteria and detection methods may lead to varying results within the same tumor type. For example, based on both typical lymphocyte aggregation with HE staining and CD20+ B-cell accumulation inside the aggregation with IHC staining, TLSs in ovarian tumors were observed by Ukita et al. in 94% of patients (17). Using LAMP+ DC and CD20+ B cells as TLS biomarkers, Truxova et al. reported less frequent TA-TLSs in OC, with a rate of 19% (14/81) (62). Lymphoid aggregates composed of B cells and T cells were observed by Kroeger and colleagues in 17 of 30 (56.67%) OC samples. However, only aggregates with prominent B-cell follicles and discrete T-cell zones were detected as fully developed TLSs (23.33%, 7/30) (38). Although IHC can largely minimize the interference caused by differences in pathologists’ experience, factors such as sample size, detection criteria, and markers used can still lead to varying results within the same type of tumor.

Based on these labor-intensive traditional quantification methods, Artificial Intelligence (AI) based image analysis techniques, including machine learning and deep learning, build on traditional quantification methods and are recognized for their potential to significantly improve the accuracy and efficiency of TLS evaluation in histopathological specimens. Recently, a plethora of deep learning algorithms have been created for the automated segmentation of TLSs across diverse malignancies, demonstrating a remarkable ability to replicate histopathologists’ evaluations with high accuracy. For example, Wang et al. broadened the applicability of these models by evaluating TLS density within lung adenocarcinoma specimens, further exploring its prognostic significance (59). Barmpoutis et al. demonstrated the successful implementation of automated TLS identification in HE stained sections through the integration of the Deep Lab v3+ architecture, active contour models, and lymphocyte segmentation techniques (60). Kushnarev et al. developed a BostonGene digital imaging analysis (DIA) platform that identifies TLS in lung cancer, demonstrating enhanced reproducibility and sensitivity compared to earlier techniques (61).Furthermore, Rijthoven et al. introduced a deep learning model called HookNet-TLS to enable the quantification of TLSs in digital pathology slides stained with HE (63). They further utilized these metrics as prognostic indicators across three distinct cancer types (clear cell renal cell carcinoma, muscle-invasive bladder cancer, and lung squamous cell carcinoma), thereby underscoring the adaptability of computational models in various oncological scenarios (64). Besides, in contrast to the studies that depended solely on the manual annotations of TLSs by pathologists with the assistance of multiplex IHC, Chen et al. developed a deep learning model for automated segmentation of TLSs with mIHC markers to precisely identify TLSs, thereby alleviating the potential biases linked to subjective human interpretation (65).Unfortunately, these innovative approaches have not yet been applied to ovarian cancer, despite their potential and technological advancements, resulting in significant gaps in research and application. Nevertheless, these advancements are vital in deepening our comprehension and application of TLSs in clinical practice.

Additionally, RNA sequencing (RNA-seq) has become an invaluable tool for the functional characterization of TLS within various tumor microenvironments. By providing a comprehensive and unbiased transcriptomic profile, RNA-seq enables the identification of key immune cell populations and signaling pathways that are active within TLS. This technology allows for the quantification of gene expression at the single-cell level, which is particularly useful in unraveling the heterogeneity of immune cells, including T cells, B cells, and dendritic cells, that orchestrate TLS function.

Due to the significant role of chemokines in TLS formation, numerous chemokine-related genes have been proposed as genetic markers for TLSs. Currently, chemokine signatures (CCL2, CCL3, CCL4, CCL5, CCL8, CCL18, CCL19, CCL21, CXCL9, CXCL10, CXCL11, and CXCL13) have been shown to accurately assess the presence of TLSs in multiple types of human tissues, including melanoma, colorectal breast cancer, and bladder cancer (66). In ovarian cancer, several genes were considered TLS-associated gene signatures. However, even with the same sequencing dataset, differences in data analysis methods and approaches might lead to differences in TLS gene signatures. For instance, using the same sequencing (TCGA-OV) and microarray data (GSE140082), Zhang and Hou obtained different OC-related TLS gene signatures. Based on transcriptome features between the TLS-high and TLS-low groups in the TCGA, Zhang et al. developed an unsupervised consensus clustering method comprising 12 chemokines to assess the relative abundance of TLSs in OC samples (5). By analyzing the TCGA-OV dataset via univariate regression, Hou et al. detected a TLS gene signature with prognostic value for ovarian cancer that included 8 genes (ETP, CCR7, SELL, LAMP3, CCL19, CXCL9, CXCL10, CXCL11, and CXCL13) (6).

**Figure 3:**
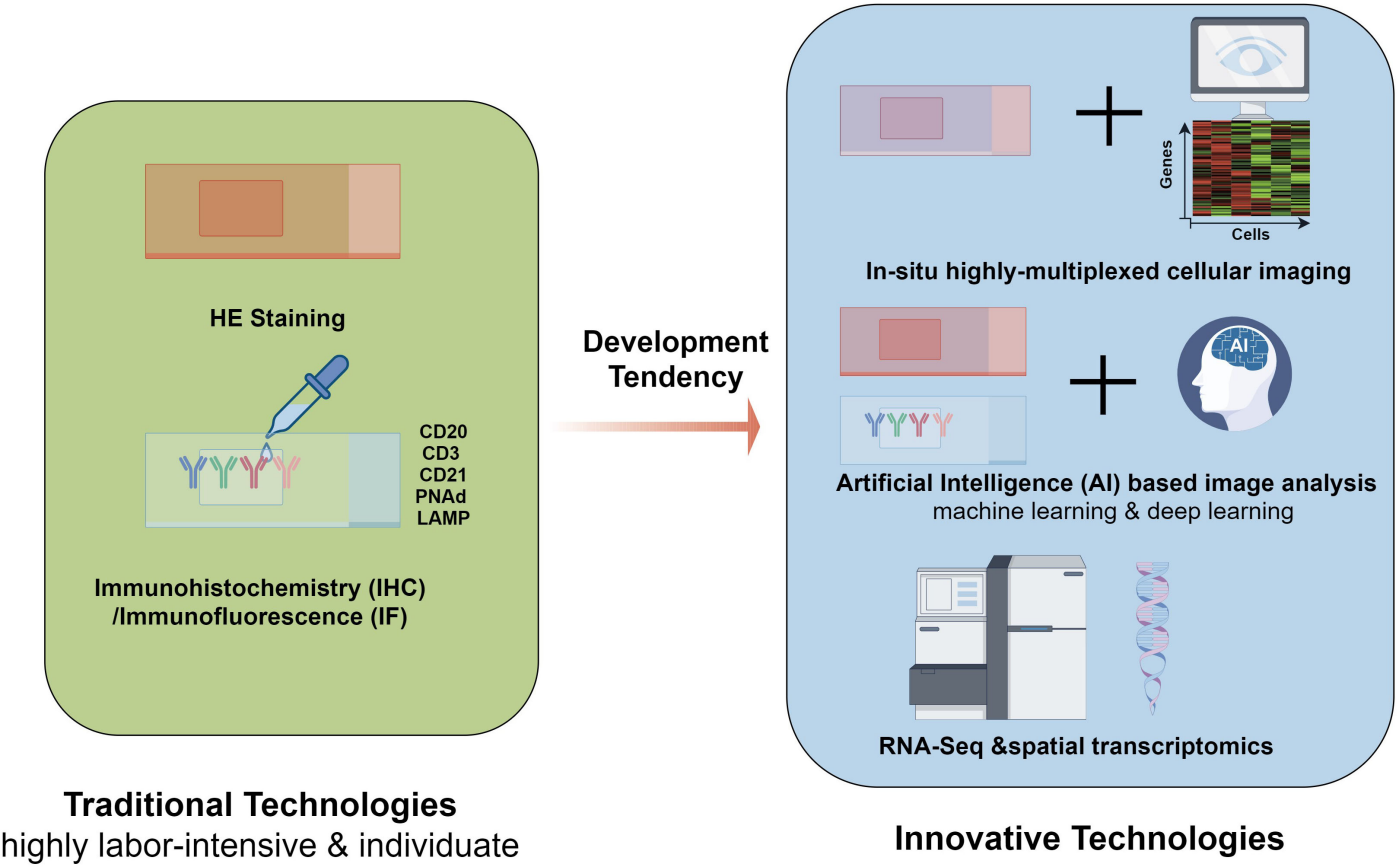
Traditional and innovative quantification technologies used in TLS evaluation. The most straightforward technology for TLSs evaluation is morphology approach-HE staining, which relies heavily on the experience of pathologists. making it labor-intensive. Immunohistochemistry (IHC) and immunofluorescence (IF) are used to achieve more solid evidence for evaluation, which makes TLS biomarkers such as CD20, CD3, CD21, PNAd, and LAMP more crucial. Based on these tranditional technologies, Artificial Intelligence (AI) based image analysis techniques, including machine learning and deep learning, are recognized for their potential to significantly improve the accuracy and efficiency of TLS evaluation in histopathological specimens. with the development of sequencing technology, innovative quantification technologies have emerged. With the extensive use of RNA-Seq and spatial transcriptome, several genes have been characterized as TLS-associated gene signatures in ovarian cancer. Furthermore, comprehensive single-cell spatial atlas of TLSs in ovarian cancer can be created by extracting spatial topology information from *in situ* highly multiplexed cellular imaging using tissue cyclic immunofluorescence, offering new insights into the spatial biology of TLSs. TLSs, tertiary lymphoid structures; IHC, Immunohistochemistry; IF, immune fluorescence; RNA-Seq, RNA sequencing.

Furthermore, the combination of spatial transcriptomics and immunohistochemistry can effectively characterize immune cell phenotypes at the gene and protein levels, both inside and outside TLS (67). Moreover, recent developments in highly multiplexed tissue technologies along with sophisticated image analysis tools, have significantly enhanced our ability to conduct more detailed and nuanced investigations of TA-TLSs at an unprecedented single-cell resolution, allowing researchers to unravel the complex cellular interactions and microenvironments that characterize these structures. Sarkkinen et al. created a comprehensive single-cell spatial atlas of TLSs in ovarian cancer by extracting spatial topology information from *in situ* highly multiplexed cellular imaging using tissue cyclic immunofluorescence, offering new insights into the spatial biology of TLSs (68). The traditional and innovative quantification technologies used in TLS evaluation mentioned above were summarized in **Figure 3**.

### 3.2 Maturity evaluation of TA-TLSs in ovarian cancer

The maturity of TA-TLSs varies dynamically within and between cancers, leading to controversy regarding the role of TA-TLSs in cancer (6, 53, 54). The presence of visible GCs on HE slides has been considered the best marker of mature TA-TLSs (44, 56, 69). As the backbone cells of the GC, FDCs are often considered biomarkers for TLSs. On this basis, a three-stage maturation classification of TA-TLSs was established for NSCLC (44), hepatocellular carcinoma (HCC) (70) and colorectal cancer (CRC) (71). Briefly, 1) AGGs are the least-organized stage and consist of ill-defined clusters of lymphocytes with neither FDCs nor segregated T and B-cell zones. 2) Primary follicle-like TLSs, which contain FDCs but without GCs, and 3) fully matured, secondary follicle-like TLSs, which should have active GCs with FDCs.

In recent studies, AGGs that had no characteristic bona fide TLSs were excluded (56, 57). The binary classification of TLSs into immature TLSs (iTLSs)/early TLSs (eTLSs) and mature TLSs (mTLSs) has been widely applied. In an OC pan-cancer study, AGGs containing more than 50 cells were detected as TLSs. However, only TLSs containing FDCs with a dendritic morphology and that were CD23+ were classified as mTLS. Notably, mature TLSs displayed prominent GCs on HE staining, which was systematically confirmed with CD23 staining (56). Based on these studies, Vanhersecke et al. proposed an algorithm suitable for screening the presence and maturity of TLSs in a variety of tumors (69). In this proposal, CD20+ B cells were the first marker of TLSs. TLSs were defined as lymphoid aggregates containing 50 nuclei of immune cells (including B cells and T cells) according to the corresponding HE staining and CD20+ according to the IHC staining.

Based on these pan-cancer studies of ovarian cancer, Ukita et al. classified TA-TLSs in HGSOC tissues into two types: 1) early TLSs (eTLSs), in which lymphocytes aggregate diffusely and CD21+ cells are scarce, and 2) follicle-formed mature TLSs, in which the follicular morphology of SLOs and CD21+ follicular DCs (FDCs) are distributed in a reticular pattern (17). Mature TLSs (mTLSs) with GCs and DCs reportedly represent privileged sites for local antigen presentation and contribute to the generation of tumor-targeting effector T cells and B cells (12).

Mature TA-TLSs have been reported to impact prognosis in several cancers, but immature TLSs play little role in antitumor immunity (53, 54). However, Hou and colleagues reported that the presence of TA-TLSs was associated with superior 5-year overall survival and progression free survival. No significant difference was observed between HGSOC patients with immature and mature TA-TLSs (6). The difference in prognosis may be due to differences in the criteria used for TA-TLS maturation. In Hou’s study, GCs and FDCs were not considered biomarkers of maturity. The evaluation of maturation relies heavily on morphological results. Small and loose aggregates of B and T cells were defined as immature TA-TLSs. The TA-TLSs with separate B-cell follicles and T-cell zones were detected as mature TLSs. GC and FDCs are not considered biomarkers of maturity (6).

Overall, the methods utilized for the detection and evaluation of TA-TLSs are primarily confined to HE and IHC, both of which have been comprehensively summarized in **Table 1**. The cellular and molecular markers that are employed to assess TA-TLSs varied widely across current studies and resulted in a broad spectrum in the reported proportion of TA-TLSs in OC (from 19%-94%). In addition to these traditional methods, several innovative technologies, such as RNA-seq, highly multiplexed tissue technologies and image analysis tools, all of which have been employed in TLS studies, offering fresh and valuable s insights into TA-TLSs of ovarian cancer.

**Table 1:** Methods for Detection and evaluation of TLSs in human ovarian cancer samples.

Sample source	Definition of TLS		TLS rate	subtypes of TLS	Ref
	HE	IHC			
HGSOC without chemotherapy	lymphocyte aggregates more than 50 cells	CD20 CD23 CD21	74% (155/209)	eTLS: aggregates of T/B cells with minimum size of 250μM, in the absence of CD21+ and CD23+ positivity FDCs (122/209 58%); mTLS: with follicles contain CD21+or CD21+CD23+ follicular DCs (33/209 16%);	(42)
HGSOC without chemotherapy	lymphocyte aggregates	CD20 CD3 CD21 PNAd	23.33% (7/30)	Type I: small lymphoid aggregate (20–50 cells), contain T cells, B cells, and occasional DCs; Type II: larger lymphoid aggregate (100-1000 cells) contain T cells, B cells, and occasional DCs diffuse but lack of discrete zones or follicles; Type III: fully developed TLS, had prominent B-cell follicles with GC-like structures (with CD21+ FDC) and discrete T-cell zones, DCs, and PNAd+ HEV	(38)
HGSOC	lymphocyte aggregates	CD20 CD3	36.67% (31/60)	iTLS: intra-tumoral loose aggregates of B and T cells (12/31 38.71%); mTLS: located in the tumor margin, with separate B cells follicles and T cells zone (19/31 61.29%);	(6)
HGSOC cases in TCGA with available H&E-stained sections	lymphocyte aggregates	CD20 CD3 DC-LAMP	39% (29/74)	iTLS: visible aggregates of immune cells in HE staining with segregated B and T cell zones; mTLS: composed of FDC, a T cell zone, and B cell follicles with a germinal center	(5)
An OC contained pan-caner cohort	lymphocyte aggregates	CD20 CD23	–	iTLS: TLS without CD23+ FDCs mTLS: TLS with CD23+ FDCs in GC;	(56)
HGSOC	lymphocyte aggregates	CD20 CD21	–	early TLS: lymphocytes aggregate diffusely with scarce CD21+ FDCs Follicle-formed TLS: follicular morphology lymphocytes aggregate with CD21+ FDCs	(17)
HGSOC omental metastases	–	CD20 CD3 MECA79	–	–	(46)
HGSOC	lymphocyte aggregates	LAMP CD20	19%	–	(62)

## 4 Cellular components of TA-TLSs and their immuno-roles in OC

### 4.1 The multifaceted roles of TLS associated B cells played

Although TLSs range from small, diffuse clusters to large, well-organized lymphoid-like structures in tumors, B cells are always integral to these structures. Several studies have suggested that in human primary tumors, B cells are mostly located in TLSs, and the presence of TLS-associated B cells is associated with a favorable prognosis in several types of cancer (22, 72–74). Tumor-infiltrating B cells bolster the antitumoral immune response in various ways, including antibody production as antibody-secreting cells (ASCs) (75) and antigen presentation to T cells as antigen-presenting cells (APCs) (76). The presence of TLS-associated B cells in ovarian cancer has been linked to a favorable prognosis (38, 46, 72) and a significant survival benefit from ICB therapy by enhancing both cellular and humoral antitumor immunity (42).

#### 4.1.1 Antibody secretion

In TA-TLSs, B cells with increased antigen affinity are selected and further transformed into memory B cells or antibody-producing plasma cells (PCs) in GCs, which are characterized as the bulk of Ki67+ proliferating B cells (46, 72, 77). PCs are often regarded as factories for antibodies and are primarily recognized for their role in humoral immunity. Some tumor-associated PCs can produce tumor-specific antibodies that bind to tumor cells. Antibodies can inhibit the activity of specific target proteins on tumor cells, activate the complement system, and enhance both antibody-dependent cellular cytotoxicity (ADCC) and antibody-dependent cellular phagocytosis (ADCP) (78).

The infiltration of PCs in ovarian cancer has a significant impact on tumor progression and prognosis (79). In HGSOCs, dense PCs, which comprise the bulk of the tumor stroma and are associated with the infiltration of CD8+ CTL cells, are frequently observed in the periphery of TLSs (38, 77). Kroeger et al. noted that the presence of PCs promoted the prognostic benefits of CD8+ CTLs in HGSOC patients. Consequently, they proposed that the coordinated antitumor responses observed in TLSs might be attributed to the synergy between cytolytic T cells and antibody-producing B cells (38). Interestingly, compared to B cells from peripheral blood, tumor-associated B cells exhibited greater levels of somatic hypermutations (SHMs), which typically occur in GCs within TA-TLSs or tumor-draining lymph nodes. Mazor et al. proved that SHMs within TLS-associated GCs enhanced the antibody response targeting surface autoantigens in HGSOC, thus bolstering antitumor reactivity. Furthermore, taking TLS-associated GCs as differentiation sites, PCs in TA-TLSs were also shown to correlate with a greater CTL response and favorable prognosis in OC patients (77).

In metastatic OC, Montfort et al. reported that the B cells in omental metastasis HGSOC samples were mainly located in TLSs. Compared with peripheral healthy B cells, the majority of these omental B cells had a “classical” (CD27+IgM+ and CD27+IgM−) or “atypical” (CD27−IgM−) memory phenotype and displayed a restricted clonal repertoire in accordance with an increased percentage of SHMs. Furthermore, in omental metastatic TLS-GC, B cells differentiate into PCs and produce tumor-targeting immunoglobulins (Igs) (46).

In ovarian cancer, infiltrating B cells frequently secrete IgG antibodies, which play a crucial role in mediating antitumor immune responses. Interestingly, with three cohorts containing 534 HGSOC patients, Anadon and colleagues found that the strong and protective humoral responses in the TME are predominantly driven by PCs producing polyclonal IgA but not by PCs producing IgG by binding to polymeric IgA receptors on ovarian cancer cells (47).

#### 4.1.2 Antigen presentation

The presence of professional APCs helps to sustain T-cell responses in the tumor environment. In addition to producing antibodies and an antibody-mediated memory response against pathogens, B cells can also generate cell-mediated immunity as APCs. Within TLSs, B cells can activate T cells through antigen-specific (BCR-dependent) and nonspecific (BCR-independent) pathways, significantly influencing immune responses in cancer (80). B cells in TA-TLSs were shown to promote the cytolytic activity of T cells and improve the survival of OC patients (22).

It has been well documented that activated B cells can present antigens to T cells (81, 82). Tumor-activated B-cell transfer has been shown to induce tumor-specific T-cell immunity in murine tumor models (83). Cabrita and colleagues showed that with highly expressed MHC I and II molecules, B cells within melanoma TLSs are generally capable of antigen presentation (73). A pan-cancer study with 237 patients demonstrated that CD21-CD86+ B cells can act as antigen-presenting B cells (BAPCs). These BAPCs are mostly found in the follicles, especially GCs, of TLSs and are important for the preservation of ICB-sensitive TCF1+PD1+CD8+ T cells in OC (84). Nielsen et al. discovered that typical markers of antigen-presenting cells, such as MHC class I/II, CD40, CD80, and CD86, were expressed on TLS-associated B cells in HGSOC. As these TLS-associated B cells are disconnected from serum autoantibodies and colocalize with CD8+ T cells, Nielsen et al. suggested that instead of producing antibodies, the predominant function of TLS-associated B cells in HGSOC is to present antigens to CD8+ T cells (45). Moreover, the degree of B-cell clonality Nielsen et al. assessed in HGSOC patients is different with that in breast cancer and germ cell tumors. In brief, 11 to 14 distinct B-cell clones were detected in HGSOC, whereas 6 to 13 clones in germ cell tumors (85), and 3 to 6 clones in breast cancer (86, 87). Similarly, the proportion of clonally derived sequences in HGSOC ranged from 58% to 66%, in comparison to 18% to 79% in germ cell tumors and 30% to 69% in breast cancer. Although these figures likely represent conservative estimates, as there may exist additional, less dominant clones that were not identified in any of these investigations. These findings still suggested that TLS associated B cells in HGSOC may exhibit a greater propensity to differentiate into APCs than other B cell subtypes. In addition, B cells can improve the CTL response in the TME by activating professional APCs such as DCs or by directly activating T cells as APCs themselves. For instance, B cells in HGSOC omental metastatic TLSs can recruit DCs by producing CXCL8 and further promote the CTL response through DC priming (46).

Generally, B cells located within TLS can play a crucial role in fostering anti-tumor immunity in ovarian cancer by not only secreting a variety of antibodies that target tumor cells but also by effectively presenting antigens to T cells, thereby enhancing the overall immune response against the malignancy.

### 4.2 T cells mediated antitumor response in TA-TLSs

In addition to B cells, T cells are also important components of TA-TLSs and are mostly located in the T zone of TLSs. Within the T zone, CD4^+^ T follicular helper (T_FH_) cells often constitute the dominant subset, and CD8^+^ cytotoxic T cells, CD4^+^ T helper 1 (T_H_1) cells, and regulatory T cells (T_regs_) can also be observed (28). Tumor-infiltrating lymphocytes (TILs) are key mediators of antitumor immunity in high-grade serous ovarian cancer (45). However, Kroeger and colleagues reported that the prognostic benefit of CD8(+) TILs in patients with ovarian cancer was limited and was only achieved in the presence of PCs, CD20+ TILs and CD4+ TILs (38). In addition, the presence of CXCL13+ CD103+ CD8+ T cells in OC was correlated with B-cell recruitment and TLS formation (88). These findings suggested that the T-cell-mediated antitumor response may require the combined actions of other lymphocyte subsets in TLSs.

Although, B cells have been proven to determine clinically relevant T-cell phenotypes in ovarian cancer. Additional studies have shown that T cells in ovarian cancer can influence the recruitment of B cells through the secretion of CXCL13, an important B-cell chemoattractant (89, 90). In TA-TLSs, the origin of CXCL13 depends on the type of cancer. T cells are the most common source of CXCL13 in TA-TLSs (88, 91). Administration of recombinant CXCL13 was reported to induce TA-TLSs and enhance survival in mouse ovarian cancer models (17, 33). In ovarian cancer, both CD8+ T cells and CD4+ T cells were observed to be the origin of CXCL13 and play important roles in mediating B-cell recruitment and TLS formation in human tumors (17, 88). Workel et al. reported that TGFβ-dependent CXCL13 secretion occurs in CD8+ T cells isolated from several human cancers, including OC (88). However, whether CXCL13 is involved in the formation of TA-TLSs and the underlying molecular mechanism are still unclear. In another study, Ukita et al. reported that CXCL13 was expressed by both T cells and DCs in human ovarian cancer. In the early stage of TLS formation, CXCL13 is predominantly expressed by CD4+ T cells. During TLS maturation, the secretion of CXCL13 transitioned from CD4+ T cells to CD21+ follicular DCs (17). However, in a study with an ovarian cancer mouse model, Ricardo et al. suggested that while the TLS-induced antitumor response is dependent on CD4+ T cells and CXCL13, CXCL13-producing T follicular helper (TFH) cells, rather than CD4+ T cells, are likely responsible for the formation of TLSs (92). The variations in these findings could be attributed to the differences in the models they used. Several studies have suggested that, unlike human CD4+ T cells, murine CD4+ T cells do not secrete CXCL13 (90, 93), which may account for the difference in these findings. In summary, these discoveries indicate that T cells in ovarian cancer, whether CD4+ or CD8+ T cells, exhibit an extraordinary capacity to activate the formation of TLS through the release of the chemokine CXCL13 and coordinate the anti-tumor immune response, thereby potentially strengthening the body’s defense mechanisms against cancer. This may present a promising therapeutic target for TLS-targeted induction therapy. Other TME Components influencing TA-TLS function.

### 4.3 The crosstalk of TLS with other cell components in TME

TLS is visually described as a T/B lymphoid aggregate that is anchored by fibroblasts and other stromal cells in sites of inflammatory microenvironment. It is crucial to recognize the importance of the interactions between these lymphoid and other TME components. In addition to T and B cells, OC-associated TA-TLSs contain myeloid cells, including DCs and macrophages. DCs are efficient antigen-presenting cells capable of capturing, processing, and presenting antigens to T cells, playing a critical role in antigen presentation and the activation of T cells, thereby enhancing the immune response within TLSs. In SLO, DCs control lymphocyte homing by producing lymphotoxin and homeostatic chemokines (94, 95). In human solid tumors, DCs are usually observed in the tumor stroma and in TLSs (96) and play a key role in TA-TLS organization by producing LT and homeostatic chemokines (97). In primary ovarian cancers, CD83+ mature DCs, which are also known as DC-LAMPs, are predominantly localized in the T-cell zone of TA-TLSs and are correlated with CD8+ T-cell infiltration, antitumor cytotoxicity and survival (62, 98). CD21+ FDCs, which are observed in B-cell follicles of TA-TLSs, are the backbone of GCs and are usually used as biomarkers of TLS maturation in OC (17, 56). Furthermore, FDCs play a key role in GC formation by producing CXCL13 (C-X-C motif chemokine ligand) and BAFF (B-Cell Activating Factor) (94, 99), both of which are dispensable for B-cell survival and function. In ovarian cancer, CD21+ FDCs are the main CXCL13 source for late-stage TA-TLS maturation and play a crucial role in the organization and maintenance of GCs (17).

Macrophages make up the largest portion of the myeloid infiltrate in most solid malignancies, including ovarian cancers (100). However, within the TME, macrophages typically present a cancer-promoting M2 phenotype, which can facilitate OC development (101). The macrophages in SLOs reportedly act as scavengers that are responsible for apoptotic cell clearance (102). Although scattered macrophages are also observed in OC-associated TA-TLSs, their exact role remains unclear. Previous studies have confirmed that macrophages can act as lymphoid tissue inducer cells or lymphoid tissue organizer cells to help inflammatory diseases associated with TLS formation (103–105). In the context of TA-TLS neogenesis, macrophages have been shown to secrete homeostatic chemokines, including CCL21 and CXCL13 (17, 106). In ovarian cancer, M1-type macrophages, but not M2-type macrophages, are one of the sources of CXCL13, which is important for TLS formation (33).

In addition to immune cells, stromal cells which are abundant and play an important role in TLSs. By providing structural support and recruiting lymphocytes, the necessity of fibroblasts in lymphoid tissue development has been extensively documented (107). Fibroblasts with LTo molecular signatures, such as VCAM-1, ICAM-1, LβR, and TNFRs I and II, can support TLS development in inflammatory diseases and tumors by expressing CCL21 or CXCL13 (29, 108). Stromal cell-biocompatible scaffolds seeded into the renal subcapsular space in mice can successfully form lymphoid tissue-like structures (109).The specialized vasculature of HEVs facilitates the entry of lymphocytes from the bloodstream into TLSs, ensuring a continuous supply of immune cells to sustain the antitumor response (51). The coordinated interaction of these cellular components is essential for the effective formation and function of TLSs in the ovarian cancer microenvironment. Fibroblasts are important supporting cells of HEVs (52). Fibroblastic stromal cells can drive tissue-specific maturation of the endothelium and support HEV angiogenesis by regulating lymphocyte recruitment and homeostasis (110, 111).

**Figure 4:**
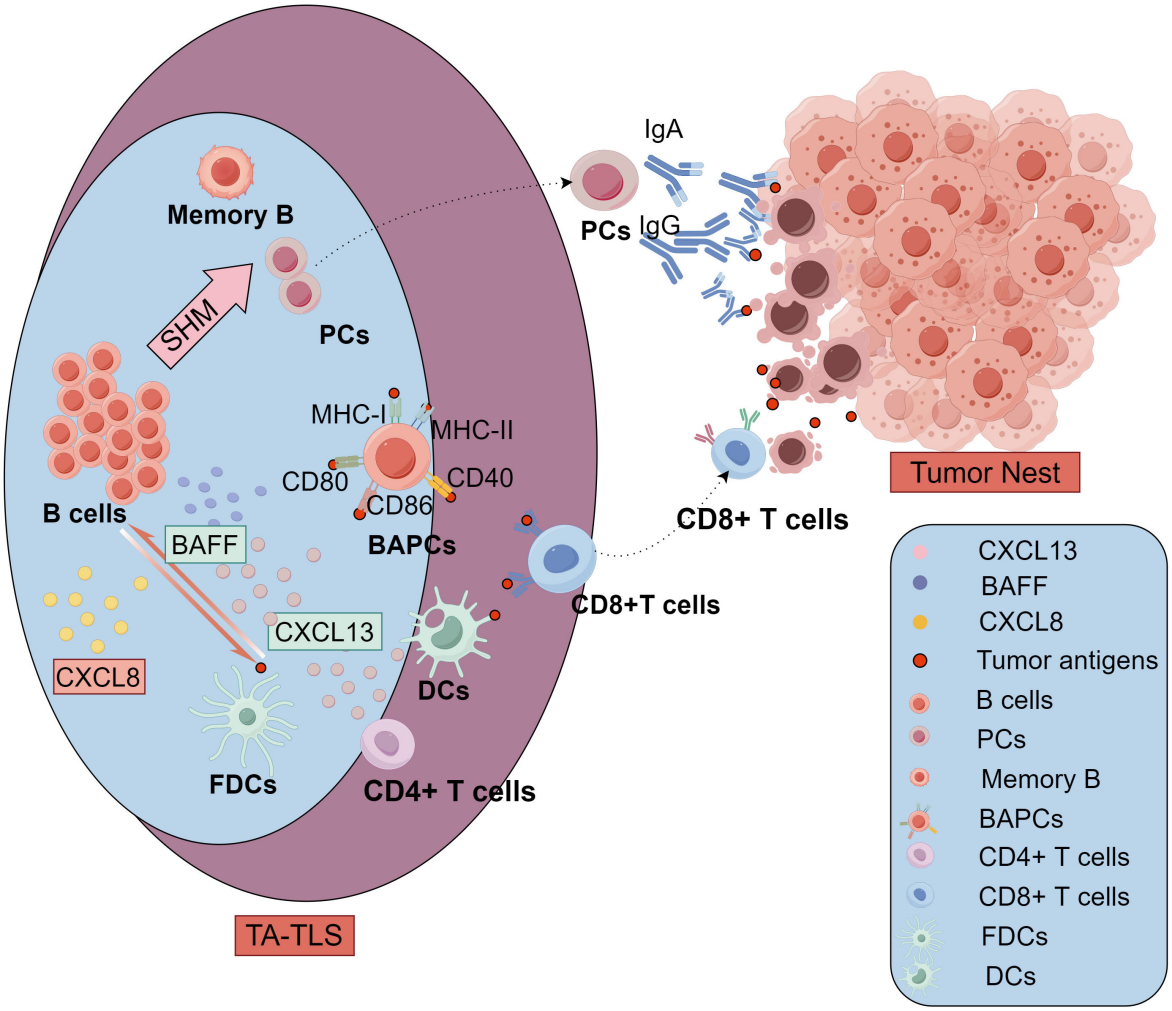
The antitumor immune responses of TA-TLSs cellular components mediated in ovarian cancer. In the B-cell region of TLSs, B cells can recruit and prime DCs through CXCL8. Meanwhile, DCs and FDCs can secrete cytokines including BAFF and CXCL13 and present tumor antigens to bolster B cell recruitment, survival, and activation. exhibited greater levels of SHMs and acquired more diverse antibody phenotypes, Activated B cells differentiate into memory B cells and PCs in GC. The PCs move to tumor nest and produce high-affinity antitumor antibodies including IgG and IgA to participate in antitumor immunity. Additionally, specialized BAPCs highly expressing typical markers of antigen-presenting cells such as MHC class I/II, CD40, CD80, and CD86, were mostly found in the follicles, especially GCs of TLSs. These non-professional APCs, BAPCs, can present tumor antigens to T cells in the TLS, just like professional APCs DCs. In the T-cell region, CD4+ T cells are one of the main sources of CXCL13 especially in early TLSs in ovarian cancer. CD8+ T cells in the TLS can be active by the antigen presentation from both DCs and BAPCs, then migrate to the tumor nest and mediate antitumor cytolytic activity. TLSs, tertiary lymphoid structures; GCs, germinal centers; FDCs, follicular DCs; BAPCs, antigen-presenting B cells; PC, plasma cells; APCs, antigen-presenting cells; DCs, dendritic cells; SHMs, somatic hypermutations, BAFF, B-Cell Activating Factor; CCL8, C-C motif Chemokine Ligand 8; CXCL13, C-X-C motif chemokine ligand 13.

Overall, there is still limited research on the crosstalk between OC-associated TA-TLSs and other TME components. We concluded the antitumor immune responses of TA-TLSs cellular components mediated in ovarian cancer in **Figure 4**. These TME components collectively contribute to the antitumor immune response in TA-TLSs. However, the heterogeneity and complexity of TLS cellular composition make standardized evaluation challenging. In particular, the flexibility of myeloid cells and the overlapping marker expression in myeloid cell and DC populations make it challenging to interpret the data. Further analysis of stromal innate immune cells such as monocytes/macrophages and DCs as initiators of cancer-associated TLSs is therefore warranted.

## 5 Induction of TA-TLSs in ovarian cancer therapy

As they serve as intra-tumoral sites where tumor-associated antigens can be consistently processed and presented (35), TA-TLSs have been described as a remarkable “antitumor school” for lymphocytes (112, 113). The evident advantages of TA-TLSs in antitumor immunology have sparked interest in exploring their possible therapeutic applications. The induction of functional TA-TLSs might be a widely applicable antitumor immunotherapy, either on their own or in combination with adoptive transfer-based cell therapies (22, 114).Although TA-TLSs can amplify local immune responses, the appearance of TLSs in untreated mice was random and required long-term inflammatory exposure (115, 116). In untreated OC patients, the presence of TA-TLSs is primarily influenced by genetic characteristics such as low-to-intermediate TMB (42), chr4q loss and DCAF15 amplification (117).

A variety of therapeutic approaches have been identified to initiate or enhance the formation of TA-TLS. For example, neo-adjuvant chemotherapy (NACT) has been demonstrated to facilitate the emergence of *de novo* TLS in NSCLC (118) and hepatoblastoma (119). In ovarian cancer, Tereza Lanickova and colleagues harnessed a diverse array of transcriptomic, spatial, and functional assays to explore the differential impacts of NACT on the progression and maturation of TA-TLS in HGSOC. Their discoveries suggest that NACT-induced endoplasmic reticulum stress, coupled with the exposure of calreticulin in metastatic HGSOC lesions, may facilitate the formation and maturation of TA-TLS and effectively maintaining an intratumoral ICI-sensitive T-cell phenotype (120). However, due to the uncertainty of therapeutically induced TA-TLSs, relevant clinical practice is still lacking. Therefore, to better leverage TLSs for therapeutic purposes, great efforts have been made in mouse models. The experimental induction of TA-TLSs in a mouse model can provide reliable preclinical data for exploring novel therapeutic mechanisms of new drugs, for example, the CDK4/6 inhibitor Abemaciclib, which has already been approved by the FDA for the treatment of breast cancer. In a mouse ovarian cancer model, Abemaciclib was reported to promote the development of TA-TLSs (121) by reducing SCD1, thereby inhibiting ATF3 and upregulating CCL4 (122). The combination of FAK knocking down and a TIGIT-blocking antibody significantly elevated CXCL13 production and the formation of TLS which lead to reduced tumor burden and increased survival in mice KMF ovarian tumor model (123).

Tumor-specific vaccines were shown to promote TA-TLS formation in cervical intraepithelial neoplasia (CIN2/3) lesions (124) and pancreatic cancer (125). However, clinical data on ovarian cancer vaccines are still limited. CpG and Mn2+ strongly stimulate LT-α and CCL21 expression in DCs to induce HEV (126, 127). Wen et al. presented a nanovaccine containing a tumor-specific antigen with CpG and Mn2+ as immunologic adjuvants. The application of this nanovaccine in a mouse tumor model successfully induced the formation of TA-TLSs and elevated local antitumor immunity (128). The alternativable tumor-specific antigen in this nanovaccine suggested its potential applications e in ovarian cancer treatment.

Since the presence of TLSs relies heavily on the expression of homeostatic chemokines such as CCL19, CCL21, and CXCL13 (129). These chemokines have been shown to initiate TLS formation even in the absence of LTi cells (130, 131). In mouse HM-1 ovarian cancer models, the administration of recombinant CXCL13 was shown to induce TA-TLSs in both abdominal metastases and subcutaneous tumor, resulting in prolonged survival (17, 33). However, different microenvironment the tumor cells seeding may result in different function of TLS. For instance, the CXCL13 induced synergy effect in anti-PD-1 therapy was observed only in subcutaneous ovarian cancer mouse model (33). It suggested that the heterogeneity of TME may significantly influences the function of TA-TLS, as evidenced by the differences in study results caused by varying tumor implantation locations.

Due to the inherent instability of recombinant cytokines, the transplantation of cells expressing homeostatic chemokines has emerged as a critical focus in research on TLS induction. As a natural source of homeostatic chemokines in TLSs, transplanted LTo-like cells have attracted increasing amounts of attention. Engraftment of tumor-derived or artificially induced LTo cells has been proven to induce TLS *in vivo (* 29, 115, 132). A recent study utilizing stromal cells derived from lymph nodes successfully established TLO *in vivo* and demonstrated that TLO induces an antitumor immune response to suppress MC38 tumor growth (115). However, the scarcity of self-stromal cells isolated from lymph nodes makes it impossible for these cells to be applied in clinical cancer treatment. Fortunately, a new study revealed an abundant and easy-to-obtain alternative. Jin et al. suggested that under the stimulation of LTα1β2 and soluble TNF-α, murine neonatal dermal fibroblasts can acquire LTO-like activity, resulting in TLS induction *in vivo* (132).

Bioengineering strategies for the fabrication of artificial LTo cells or micro-TILs *in vitro*, aiming to enhance the adaptive immune response, will offer promising therapeutic applications in cancer immunotherapy. Intrapulmonary administration of CCL21 gene-modified DCs has been shown to effectively induce TA-TLSs and reduce the tumor burden in spontaneous murine bronchoalveolar cell carcinoma (133). Moreover, Sachiko and colleagues engineered LTα overexpressed TEL-2 thymic stromal cells (TEL-2-LTa). After transplanting TEL-2-LTa into the renal subcapsular space in mice using collagen scaffolds, typic TLS (109), and secondary immune responses *in vivo* were obseved (134). Adipose stromal vascular fraction cells (SVFs) which have phenotypes and functions similar to fibroblastic in SLO were 3D spheroid cultured. then Lee et al. cocultured the SVF 3D spheroid with DCs spiked with antigen-loaded Fe3O4–ZnO Core-Shell NPs to form a cell loaded scaffold which offered a distinct niche for DCs to promote T cell recruitment and the subsequent TLS establishment *in situ* (135). Furthermore, Wang et al. reported a tissue bioengineering approach to rapidly synthesize human HEV organoids (HEVOs) using human induced pluripotent stem cells (hiPSCs) with the instructions of FRCs but not DCs (111). The implantation of these HEVOs successfully induced TLS formation and an adaptive immune response in a mouse tumor model (111). While these innovative initiatives have yet to be implemented in OC, they also offer a potential approach for the investigation of TLS induction therapy in OC.

## 6 Discussion

In the setting of cancer, TLSs are receiving increased attention because they have been associated with favorable prognosis in several solid tumors, including ovarian cancer. The correlation between TLS presence and improved clinical outcome in patients with OC suggested that the TA-TLS could serve as a valuable prognostic marker, offering insights into patient outcomes and guiding therapeutic decisions. The formation of TLSs in ovarian cancer is orchestrated by a complex interplay of chemokines, cytokines, and various cellular components influenced by genetic and epigenetic factors, which underscores the dynamic nature of the tumor microenvironment. From a therapeutic standpoint, targeting TLSs to enhance antitumor immunity represents a promising avenue. Modulating TLS formation and function with administration of chemokines (17, 33), chemotherapy or immunotherapy agents (121, 123) have significantly amplify the immune response and survival outcomes in mice HGSOC model and offering a novel strategy to complement existing therapies. The integration of TLS-targeting approaches with conventional treatments, such as chemotherapy and immunotherapy, could lead to synergistic effects, improving overall treatment efficacy.

However, Researches have indicated that the composition and function of TLS were differ between various tumors and even within a single tumor (136). Conducting comprehensive analyses that compare ovarian cancer-associated TLSs with those in other cancer types is very important, as it may allow for a thorough evaluation of TLS heterogeneity. While TA-TLS has been recognized and studied in nearly all tumor types, it is important to note that much of the existing data comes from studies that use inconsistent markers to define TLS. Until now, comprehensive analyses using a uniform set of parameters have been absent. In a previous study, Kasikova et al. compared the TA-TLSs in immunologically “cold” tumors HGSOC with immunologically “hot” tumors NSCLC using uniform TLS biomarkers (42). Their evidence indicated that TLSs in HGSOC are not only less frequent but also less developed than NSCLC. Specifically, TLSs in HGSOC have a significantly lower density of CD4+ T cells, GZMB+CD4+ T cells, GZMB+CD8+ T cells, CD4+CXCR5+PD1+FoxP3− TFH cells, and especially follicular DCs. Furthermore, while the frequency of TIM3+PD1+CD8+ T cells was similar in HGSOC and NSCLC samples, PD1+CD8+ T cells in NSCLC were more likely to polarize into an ICB-sensitive TCF1+PD1+ phenotype with effector functions. In contrast, the density of CD68+ TAMs and PD1−FoxP3+CD4+ regulatory T (T_regs_) cells was similar in the mTLSs of both HGSOC and NSCLC samples. In conclusion, they conducted a thorough comparison of TA-TLSs in HGSOC and NSCLC with a same detailed molecular panel. These findings enhance our understanding of the complex roles of TLS in both cancers and pave the way for future research and potential therapies.

Furthermore, Sarkkinen and colleagues show that the immune function especially active adaptive immunity in TLSs varies among the clinical and molecular subtypes of HGSOC, based on analyses of TLSs using single-cell techniques (68). This underscores the importance of examining the diversity within ovarian cancer. Compared to HGSOC, other subtype of ovarian cancer, such as mucinous ovarian cancer, which is deficiency immunogenically ‘cold’ (137) and with low mismatch repair (138), have been reported to differ significantly in their immune microenvironment. As a result, TA-TLS may vary significantly among the various subtypes of ovarian cancer. However, TA-TLSs in ovarian cancer are most frequntly doumcumented in HGSOC. This emphasis on HGSOC creates a significant gap in our understanding of TLS in other types of ovarian cancers, leaving a crucial area of research unexplored.

Moreover, the intricate journey toward harnessing TLSs in ovarian cancer is fraught with numerous challenges. While the induction of TLSs holds significant promise for enhancing antitumor immunity and potentially improving patient outcomes, it also poses potential risks and side effects. One major concern is the possibility of inducing autoimmunity or exacerbating existing autoimmune conditions in patients who may already be vulnerable. Since TLSs can form in response to chronic inflammation, their induction in noncancerous tissues could lead to unintended immune activation against self-antigens, thereby triggering autoimmune responses. Although limited data exist on the role of TLSs in immune-related adverse events, an association between TLS formation and autoimmune myopathy upon PD-1 blockade has been reported (139). Moreover, systemic administration of chemokines and cytokines to promote TLS formation could result in off-target effects, causing inflammation and damage to healthy tissues, which could complicate the clinical picture. Another potential side effect is the alteration of the tumor vasculature, which could impact the delivery and efficacy of other therapeutic agents. Thus, while the induction or enhancement of TLSs may boost antitumor responses, and offer new avenues for treatment, it could also expand autoreactive T and B cells, necessitating a careful evaluation of the risk–benefit ratio of such approaches.

In conclusion, the intricate and complex role of TLSs in the context of ovarian cancer presents both significant opportunities for research and considerable challenges in advancing our comprehensive understanding and effective treatment of this malignancy. This review underscores the multifaceted nature of TLSs, highlighting their formation mechanisms, and the intricate processes involved but also their profound impact on tumor progression and patient prognosis, as well as their promising potential for therapeutic applications that could revolutionize current treatment strategies.
